# Effectiveness of a Web-Based Health Education Program to Promote Oral Hygiene Care Among Stroke Survivors: Randomized Controlled Trial

**DOI:** 10.2196/jmir.7024

**Published:** 2017-03-31

**Authors:** Normaliza Ab Malik, Sa'ari Mohamad Yatim, Otto Lok Tao Lam, Lijian Jin, Colman Patrick Joseph McGrath

**Affiliations:** ^1^ Periodontology and Dental Public Health The University of Hong Kong Hong Kong SAR China; ^2^ Faculty of Dentistry Universiti Sains Islam Malaysia (USIM) Kuala Lumpur Malaysia; ^3^ Hospital Serdang Department of Rehabilitation Medicine Selangor Malaysia; ^4^ Department of Oral Rehabilitation The University of Hong Kong Hong Kong SAR China

**Keywords:** oral hygiene, computer-aided learning, cerebrovascular accident, theory of planned behavior, health care providers, Internet

## Abstract

**Background:**

Oral hygiene care is of key importance among stroke patients to prevent complications that may compromise rehabilitation or potentially give rise to life-threatening infections such as aspiration pneumonia.

**Objective:**

The aim of this study was to evaluate the effectiveness of a Web-based continuing professional development (CPD) program on “general intention” of the health carers to perform daily mouth cleaning for stroke patients using the theory of planned behavior (TPB).

**Methods:**

A double-blind cluster randomized controlled trial was conducted among 547 stroke care providers across 10 hospitals in Malaysia. The centers were block randomized to receive either (1) test intervention (a Web-based CPD program on providing oral hygiene care to stroke patients using TPB) or (2) control intervention (a Web-based CPD program not specific to oral hygiene). Domains of TPB: “attitude,” “subjective norm” (SN), “perceived behavior control” (PBC), “general intention” (GI), and “knowledge” related to providing oral hygiene care were assessed preintervention and at 1 month and 6 months postintervention.

**Results:**

The overall response rate was 68.2% (373/547). At 1 month, between the test and control groups, there was a significant difference in changes in scores of attitude (*P*=.004) and subjective norm (*P*=.01), but not in other TPB domains (GI, *P*=.11; PBC, *P*=.51; or knowledge, *P*=.08). At 6 months, there were significant differences in changes in scores of GI (*P*=.003), attitude (*P*=.009), SN (*P*<.001) and knowledge (*P*=.001) between the test and control groups. Regression analyses identified that the key factors associated with a change in GI at 6 months were changes in SN (beta=.36, *P*<.001) and changes in PBC (beta=.23, *P*<.001).

**Conclusions:**

The Web-based CPD program based on TPB increased general intention, attitudes, subjective norms, and knowledge to provide oral hygiene care among stroke carers for their patients. Changing subjective norms and perceived behavioral control are key factors associated with changes in general intention to provide oral hygiene care.

**Trial Registration:**

National Medical Research Register, Malaysia NMRR-13-1540-18833 (IIR); https://www.nmrr.gov.my/ fwbLoginPage.jsp

## Introduction

Provision of oral hygiene care is often underemphasized and underpracticed in the acute hospital setting [1]. This is despite growing acceptance of the importance of oral hygiene to general health, because of its potential link with bacteremia and aspiration pneumonia [2,3]. For stroke patients in the acute hospital setting, it is recognized that oral hygiene care is of key importance to prevent complications that may compromise rehabilitation or potentially give rise to a recurrent stroke [4]. There is a growing interest in how to effectively increase the practice of providing oral hygiene care in the hospital setting through clinical interventions [5,6] and through education and training of caregivers [7,8]. Unfortunately, however, all this has met with limited success and a persistence of poor knowledge and attitudes toward providing oral hygiene care in the acute hospital setting typically prevails [9].

Despite the acknowledged importance of dental education and oral health promotion activities, the effectiveness to change practices with respect to oral hygiene care has met with limited success among carers and patients [10,11]. To this end, the need to plan and implement oral health promotion programs based on psychological models has advocated the need to “translate theory into practice” [12]. One of the most widely used theories (model) is the theory of planned behavior (TPB), which emphasizes the importance of changing the “general intention”’ (GI) to perform a health behavior and its relationship to attitude (positive or negative views of a behavior), subjective norm (SN, perceived of social pressure to perform a behavior) and perceived behavior control (PBC, one’s control to perform a behavior) [13,14].

Providing dental health education and oral health promotion through continuing professional development (CPD) programs is an important and practical way to promote oral hygiene care practices in hospital settings [15]. The use of Web-based and computer-aided learning (CAL) has been widely used in CPD programs for health carers, owing to its ability to implement programs across wide geographical areas at relatively low costs and because of the reported effectiveness of such programs in changing health care practices [16-20] and health behavior [21]. Increasingly, Web-based and CAL programs are being used to enhance oral hygiene care in hospital and other institutionalized setting, and there are several reports of their ability to positively bring about change in knowledge [22], attitudes [18], and oral health behavior [23].

We aimed to evaluate the effectiveness of a Web-based (CPD) program to change the GI of health carers to perform daily mouth cleaning for stroke patients using TPB in a large randomized controlled trial across Malaysia. In addition, the study aimed to identify key factors associated with changes in GI among health care workers to provide oral hygiene care to stroke patients.

## Methods

### Study Design and Sample

This study was a double-blind, cluster-randomized, controlled trial with 1 month and 6 months follow-ups. The study involved 10 public hospitals in Malaysia, which have participated in a survey of oral hygiene practice for stroke patients. These hospitals were selected because they provide rehabilitation services that are led by rehabilitation medicine specialists. Hospitals were first stratified by size into either large, medium, or small in terms of number of health care providers. From each stratified group, hospitals were block-randomized in groups of 4 (“ABBA”) by a computer-generated randomization method. In total, 5 hospitals were assigned to the test group (277 registered nurses) and 5 hospitals were assigned to the control group (270 registered nurses). The allocation sequence was concealed from the investigator coordinating the trial (who had contact with the centers). Through concealment, the assessor was “blind” as to what group participants had been assigned and participants were also blind as to what groups they were assigned to, as both received a form of Web-based CPD.

The study population was registered nurses caring for stroke patients at the hospitals, mainly from the rehabilitation and general medical wards. All the registered nurses from these identified wards were invited to take part in this trial. Information sheet related to the study and written informed consent were given to all the nurses before commencing the study. The forms were distributed to the nurses by the ward managers or chief nurses. Nurses who provided their written consent were those who participated in the trial from the study population. Participation was voluntary and no contact was made with the nurses to ensure confidentiality and reduce the potential for “social bias.”

### Ethics Approval

This clinical trial was registered with the National Institutes of Health, Ministry of Health, Malaysia; NMRR-13-1540-18833(IIR). Before the commencement of the study, ethical approval was obtained from the Institute for Health Behavioral Research and Medical Research and Ethics Committee of the National Institutes of Health, Ministry of Malaysia. Permissions to conduct the study were also obtained from the directors of the respective hospitals. Recruitment and baseline assessments were from September 2014 to November 2014 at 10 hospitals across the country (both the Peninsular Malaysia and island of Borneo Malaysia). This study followed CONSORT guidelines.

### Data Collection

The nurses self-completed a questionnaire on the practice of providing oral hygiene care to stroke patients, which contained 12 items specific to attitudes, SN, PBC, and GI to providing oral hygiene care related to TPB. These items were derived from the manual of “Constructing Questionnaires Based on the Theory of Planned Behavior” developed by the Centre of Health Services Research, University of New Castle, UK (2004) [24]. Items related to direct measure of the domains were chosen and modified to the oral health context. For example, in the GI domain, “I expect to measure the blood pressure of my patients with diabetes in each consultation” was modified to “I expect to perform oral care (including denture) for patients in every session.” Each domain had 3 items that were rated on a 5-point Likert scale (strongly disagree, disagree, not disagree or agree, agree, and strongly agree). Domain scores can range from 3 to 15, with higher scores reflecting more positive attitude, stronger subjective norms, greater perceived behavior control, and greater general intention to provide oral hygiene care. Sociodemographic and environmental characteristic (eg, attended oral care training, availability of oral health guidelines and oral hygiene kits, and having dental professional support in the ward) information were also obtained from the participants.

In addition, knowledge of oral health care was assessed using 5 items related to dental plaque, gum bleeding, consequences of dental plaque, how to prevent gingivitis, and how oral health affects general health [25]. Knowledge scores can range from 0 to 5, with higher scores indicative of greater oral health knowledge. Assessments were carried out preintervention and at 1 month and 6 months postintervention.

### Intervention

A Web-based CPD program was developed for the test and control groups. The test group program was specific to provision of oral hygiene care to stroke patients and covered details of oral health knowledge, attitudes, subjective norms, means of behavioral control, and intention (ie, based on TPB). The test group contents include, for example, information on good oral condition and the importance of having good oral health, the consequences of poor oral hygiene, and the importance of nurse’s roles and care of stroke patients. The development of the contents was guided by the definition of the TPB domains and scope of the study. The control group received an analogous Web-based CPD program related to “bundles of care” for stroke patients that included some details on oral hygiene care but not specific to TPB [26]. The CPD programs were developed by stroke physicians (rehabilitation medicine) and dentists and followed good practices of CAL for oral health [27]. Following the assignment to the groups, the participants were provided with details of the Web-based programs through a secure internet portal. Participants were reminded and encouraged to complete the Web-based CPD program every 6 weeks.

### Sample Size

With the assumption that this practice is at 50% and that it will not change without education intervention, whereas there will be a 25% improvement in practices following CAL intervention (ie, 63% of nurses will practice oral care in rehabilitation). Then a proposed sample size of 247 in each group is required with sample power at 80%. Allowing for nonparticipation and a dropout rate of ~20%, thus it was prudent to attempt to recruit over 600 nurses (300 per group) in total to test the hypothesis.

### Data Analysis

The changes in knowledge, attitudes, SN, PBC, and GI were determined overtime and compared between the test and control groups using Friedman two-way analysis of variance (ANOVA) and Mann-Whitney *U* test analysis, respectively. Multiple linear regression analyses were performed to determine key factors associated with changes in GI to provide oral hygiene care at 1 month and 6 months.

## Results

**Figure 1:**
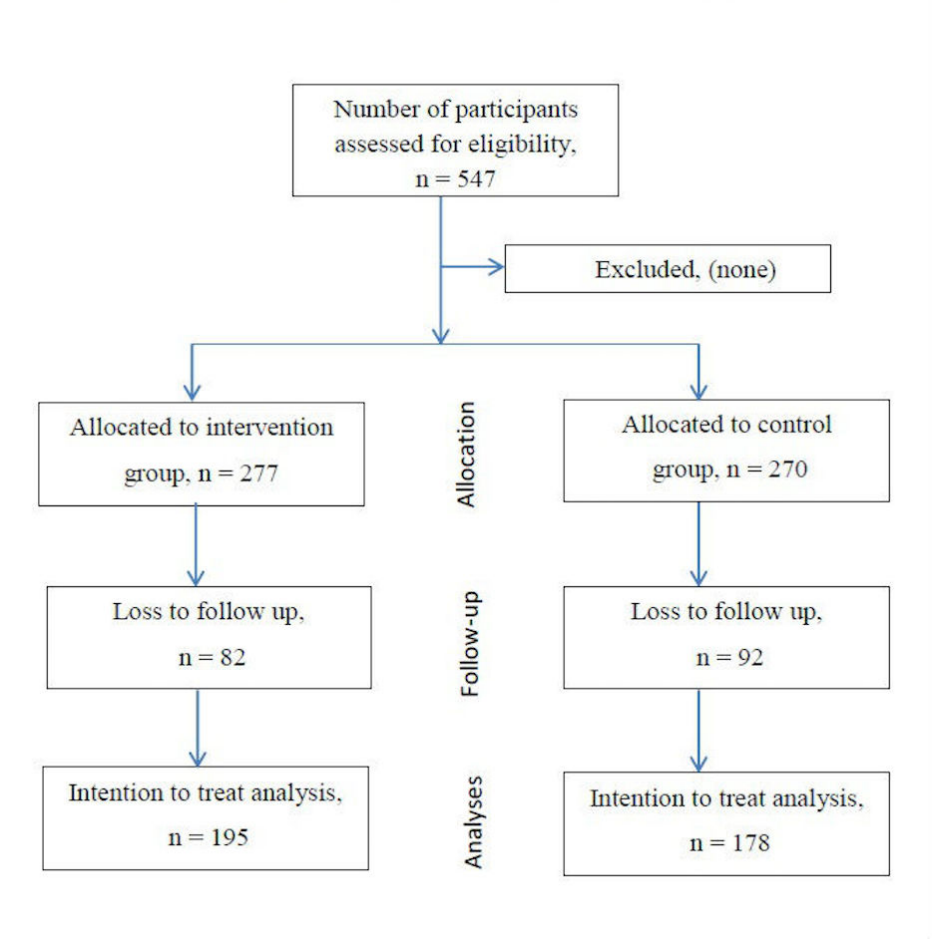
Flow diagram phases of the two-group randomized controlled trial.

The response rate of the trial was 68.2% (373/547); mostly loss to follow-up was because nurses were transferred to other wards or hospitals (Figure 1). The response rate among the test group was 70.4% (195/277) and among the control group was 65.0% (178/270); there was no significant difference between the response rate among those in the test and control groups (*P*>.05). The majority of nurses were female (95.7%, 357/373), had a certificate or diploma in nursing (81.5%, 304/373), worked in general medical wards (78.6%, 293/373), and reported to have worked for less than 5 years (59.0%, 220/373; Table 1).

**Table 1:** Health care provider and environmental characteristics (n=373)

Characteristics			n (%)
Provider characteristics			
	**Gender**		
		Male	16 (4.3)
		Female	357 (95.7)
	**Years worked**		
		Less than 5 years	220 (59.0)
		More than 5 years	153 (41.0)
	**Qualification**		
		Certificate or diploma	304 (81.5)
		Post basic or degree	69 (18.5)
	**Working wards**		
		Rehabilitation ward	80 (21.4)
		Medical ward	293 (78.6)
Environmental				
	**Oral care training**		
		Yes	108 (29.0)
		No	265 (71.0)
	**Oral health care guidelines**		
		Yes	296 (79.4)
		No	77 (20.6)
	**Oral hygiene kit**		
		Yes	253 (67.8)
		No	120 (32.2)
	**Dental professional support**		
		Yes	52 (13.9)
		No	321 (86.1)

Among all participants, there was a significant difference in knowledge scores over time (*P*<.05; Table 2). There was a significant improvement in knowledge scores between baseline and 1 month (*P*<.01), but no significant change between baseline and 6 months (*P*>.05), and between 1 month and 6 months (*P*>.05).

**Table 2:** Changes in knowledge and theory of planned behavior domains scores over time.

Time	General intention Mean (SD)	Attitudes Mean (SD)	Subjective norm Mean (SD)	Perceived behavior control Mean (SD)	Knowledge Mean (SD)
Baseline	10.8 (2.1)	13.1 (1.7)	9.7 (1.7)	10.5 (1.7)	3.0 (1.0)
1 month	11.0 (1.9)	12.9 (1.6)	9.9 (1.7)	10.3 (1.9)	3.2 (1.1)
6 months	10.7 (2.2)	13.0 (1.6)	9.7 (1.8)	10.3 (1.9)	3.2 (1.1)
*P* value	.36	.11	.05	.28	.005^a^

^a^Baseline<1 month, *P*=.02.

In the test group, there was a significant difference in knowledge scores over time (*P*<.001; Table 3). There was a significant improvement in knowledge scores between baseline and 1 month (*P*<.01) and between baseline and 6 months (*P*<.01), but no significant change between 1 month and 6 months (*P*>.05). There was a significant change in GI scores between baseline and 1 month (*P*<.05). A significant improvement in SN scores was observed between baseline and 1 month (*P*<.05) and between baseline and 6 months (*P*<.01), but no significant change between 1 month and 6 months (*P*>.05). However, no significant changes over time were observed in attitude scores (*P*>.05) and PBC scores (*P*>.05).

**Table 3:** Test and control group changes in knowledge and theory of planned behavior (TPB) domain scores overtime.

Group		General intention Mean (SD)	Attitude Mean (SD)	Subjective norm Mean (SD)	Perceived behavior control Mean (SD)	Knowledge Mean (SD)
**Test group**							
	Baseline	10.6 (2.0)	12.9 (1.6)	9.5 (1.7)	10.5 (1.7)	2.9 (1.0)
	1 month	11.0 (2.0)	12.9 (1.8)	10.0 (1.8)	10.2 (2.1)	3.3 (1.1)
	6 months	10.9 (1.9)	13.2 (1.6)	10.1 (1.5)	10.2 (1.9)	3.3 (1.1)
	*P* value	.045^a^	.43	.001^b^	.42	<.001^c^
**Control group**							
	Baseline	11.0 (2.1)	13.3 (1.8)	9.8 (1.8)	10.6(1.7)	3.0 (1.0)
	1 month	11.0 (1.9)	12.8 (1.5)	9.7 (1.6)	10.5 (1.6)	3.1 (1.0)
	6 months	10.6 (2.5)	12.9(1.7)	9.3 (2.0)	10.4 (1.8)	3.0 (1.1)
	*P* value	.03	.002^d^	.22	.57	.16

^a^Baseline<1 month, *P*=.03.

^b^Baseline<1 month, *P*=.02; Baseline<6 months, *P*=.004.

^c^Baseline<1 month, *P*=.009; Baseline<6 months, *P*=.003.

^d^Baseline>1 month, *P*=.01.

Among the control group (Table 3), there was a significant change in GI scores (*P*<.05) and attitude scores (*P*<.01), no significant changes between baseline, 1 month, and 6 months, and significant changes between baseline and 1 month with lower score at 1 month (*P*<.05), respectively. No significant changes over time were observed in SN scores (*P*>.05), PBC scores (*P*>.05), and knowledge scores (*P*>.05) among the control group.

At 1 month, between the test and control groups, there were significant differences in the change (∆) of attitude scores (*P*<.01) and SN scores (*P*<.05), but not in other TPB domains (*P*>.05) nor in knowledge scores (*P*>.05; Table 4). At 6 months, between the test and control group, there were significant differences in the change (∆) of GI scores (*P*<.01), attitude scores (*P*<.01), and SN scores (*P*<.001), but not in PBC (*P*>.05). In addition, there were significant differences in the change of knowledge scores (*P*<.01).

**Table 4:** Changes in knowledge and theory of planned behavior domain between test and control groups from baseline to 1 month and baseline to 6 months.

Time		∆GI	∆Attitude	∆SN	∆PBC	∆Knowledge
		Mean (SD)	Mean (SD)	Mean (SD)	Mean (SD)	Mean (SD)
**1 month**						
	Test	.42 (2.57)	.01 (2.23)	.45 (2.18)	−.33 (2.68)	.39 (1.37)
	Control	.02 (2.61)	−.47 (2.21)	−.13 (2.45)	−.07 (2.11)	.08 (1.46)
	*P* value	.11^a^	.004^a^	.01^a^	.51^a^	.08^a^
**6 months**						
	Test	.29 (2.45)	.24 (2.01)	.54 (2.16)	-.33 (2.51)	.41 (1.36)
	Control	−.40 (2.94)	−.35 (2.42)	−.51 (2.71)	−.12 (2.44)	−.08 (1.46)
	*P* value	.003^a^	.009^a^	<.001^a^	.51^a^	.001^a^

^a^*P* values derived from the Mann–Whitney *U* test.

Findings of the regression analyses are presented in Table 5. At 1 month, predictors of GI were attitude at 1 month (beta= .40, *P*<.001) and SN at 1 month (beta= .40, *P*<.001). At 6 months, predictors of GI were SN at 6 months (beta= .43, *P*<.001) and PBC (beta= .29, *P*<.001). Predictors of ∆ in GI scores between baseline and 1 month were ∆ (between baseline and 1 month) in attitude (beta= .31, *P*<.001) and ∆ (between baseline and 1 month) in SN (beta= .34, *P*<.001). Predictors of ∆ in GI scores between baseline and 6 months were ∆ (between baseline and 6 months) in SN (beta= .36, *P*<.001) and ∆ (between baseline and 6 months) in PBC (beta= .23, *P*<.001; Table 6).

**Table 5:** Multiple linear regression analyses to predict general intention to provide oral care at 1 month and 6 months.

	Model 1 (1 month)			Model 2 (6 months)			
Items	B^a^	SE^b^	*P* value		B^a^	SE^b^	*P* value
Group	−.16	.18	.38		−.01	.21	.98
Knowledge at 1 month	.01	.08	.96		.06	.09	.49
TPB^c^							
Attitude at 1 month	.40	.05	<.001		.10	.06	.12
SN^d^ at 1 month	.40	.05	<.001		.43	.06	<.001
PBC^e^ at 1 month	.05	.05	.34		.29	.05	<.001
	R^2^-adjusted model =.23			R^2^-adjusted model 1=.22			

^a^B: parameter estimate.

^b^SE: standard error.

^c^TPB: theory of planned behavior.

^d^SN: subjective norm.

^e^PBC: perceived behavior control.

**Table 6:** Multiple linear regression analyses to predict changes in general intention between baseline and 1 month, and baseline and 6 months to provide oral care

	Model 3 (Baseline and 1 month)			Model 4 (Baseline and 6 months)		
Items	B^a^	SE^b^	*P* value	B^a^	SE^b^	*P* value
Group	.07	.25	.79	.30	.26	.25
∆^f^Knowledge	.01	.09	.96	.01	.09	.96
TPB^c^						
∆Attitude	.31	.06	<.001	.10	.06	.09
∆SN^d^	.34	.05	<.001	.36	.05	<.001
∆PBC^e^	.07	.05	.21	.23	.05	<.001
	R^2^-adjusted model =.20			R^2^-adjusted model 1=.21		

^a^B: parameter estimate.

^b^SE: standard error.

^c^TPB: theory of planned behavior.

^d^SN: subjective norm.

^e^PBC: perceived behavior control.

^f^∆ denotes change.

## Discussion

### Principal Findings

As mentioned before, there is a need to improve the practice of oral hygiene care in the acute hospital setting, and some would argue particularly for stroke patients [28-31]. CAL has been used widely to deliver information and educate patients and caregivers in the medical field but few were reported to be related to oral health [32,33]. Thus, this study aimed to evaluate the effectiveness of a Web-based (CPD) program to change the GI of health carers to perform daily mouth cleaning for stroke patients using TPB and to identify the key factors associated with changes in GI among health care workers to provide oral hygiene care to stroke patients. Increasingly, there are calls to consider theory-based health promotion strategies in implementing programs and to provide evidence of whether theory does, in fact, dictates practice [34,35].

This study provided support for the application of TPB among stroke carers to perform oral care daily. Over time, in the test group, there was evidence of significant improvements in general intentions to perform oral hygiene care, SN, and knowledge. Even by 1 month, there was a significant improvement in GI, SN, and knowledge, and between 1 month and 6 months this persisted. Whereas in the control group there was a significant change in GI and attitudes, but it was transient; a significant difference was between 1 month and baseline but not between 6 months and baseline. At 1 month, there were significant differences in the change of attitude scores and in the change of SN scores between the test and control groups. By 6 months, significant differences in the change in GI, attitudes, and SN to provide oral hygiene care were evident in all TPB domains except in PBC. In addition, a significant difference in the change of knowledge scores over the 6-month period was also evident between the test and control groups. This provides evidence of the effectiveness of the Web-based intervention and suggests that its effectiveness increases over time. Nonetheless, PBC did not significantly change. It is plausible that this may take longer to change or needs to be supported by environment changes [36].

Regression analyses identified that the key factors associated with GI (to provide oral hygiene care) at 1 month were attitudes (at 1 month) and SN (at 1 month); and that general intention at 6 months was associated with SN (at 6 months) and PBC (at 6 months). Furthermore, the change in GI (between baseline and 1 month) was associated with the changes that occurred in attitudes and SN; and the change in GI (between baseline and 6 months) was associated with the changes that occurred in SN and PBC. The fact that several of TPB domain scores were associated with GI at the respective time periods and that the changes in several of the TPB scores were associated with changes in GI at the respective periods provide evidence of the interrelationship between domains of TPB and GI as the theory hypothesizes. The findings of this trial support other findings with respect to predictors of GI at specific time periods [37] and changes in GI [13]. Thus in line with TPB, carers who had positive attitudes, perceived positive social pressure, and were in control of their action would have high intention of performing oral care daily to patients with stroke [13]. Of note, some domains did not change nor were some domains associated with GI or change in GI, thus the influence of the domains varies across the study [37]. This is not an unusual feature in studies as not all domain changes are associated with GI over time [38,39]. This perhaps can be attributed to the context-specific settings of the study, the type of intention, and indeed the general intention practice [40,41].

Studies have shown that a significant improvement in GI, SN, and knowledge among the participants increased their confidence in promoting oral health and educating patients [19]. A noteworthy finding of this study was that both groups were given the CAL program, but only the intervention group had detailed information on oral health related to stroke patients. Thus this contributed to a significant increase in specific knowledge and GI among the intervention group compared with the control group [18].

The study benefits from its relatively large sample size, the diverse geographical areas within a country (major centers for stroke rehabilitation), and being a double-blind randomized controlled clinical trial. The response rate was close to 70% and loss to follow-up was largely attributed to nurses having changed working environments because of being assigned to other wards or hospitals. There was no significant difference in response rate between the test and control groups.

In this trial, participants were block-randomized by hospital (stratified by size) rather than by participants to avoid the potential of bias of participants discussing the Web-based CPD program within centers. The use of Web-based CPD in both the test and control group interventions allowed for a double-blind trial. In the test group, the program was built on TPB and included specific information to promote increases in knowledge, attitudes, SN, PBC, and GI to provide oral hygiene care. Although the control group program did contain some information on oral hygiene care, it was limited and not based on the TPB.

Going forward, it would be worth investing if changes in the practice of provision of oral hygiene care do occur and that the practice is maintained over time. Furthermore, studies can be done to examine the pathways that influence changes in behavior by considering specific beliefs of TPB attributes and the impact of Web-based CPD. The impact of CPD depends on various factors such as individual carer characteristics (eg, personal compliance or interest to update themselves with new information) [42,43], and environmental factors (eg, lack of Internet access or software compatibility) [22,44]. It would also be useful to monitor engagement in the Web-based learning (which at the time keeping anonymity) to determine how this effects outcomes or indeed other methods of learning.

### Conclusions

A Web-based CPD program based on TPB was effective in increasing GI to perform oral hygiene care for stroke survivors in the acute hospital setting. In addition, the program was effective in changing attitudes and SN. Furthermore, the study found that changing SNs and PBC are key factors associated with changes in GI to provide oral hygiene care. These findings support and have implications for the use of theory-based health education CPD programs and oral health promotion programs.

## Abbreviations

CAL: computer-aided learning
CPD: continuing professional development
GI: general intention
PBC: perceived behavior control
TPB: theory of planned behavior
SN: standard norm
